# P-358. Long-Acting Cabotegravir and Rilpivirine in People Living with HIV and Obesity: Real-World Insights from the RELATIVITY Cohort

**DOI:** 10.1093/ofid/ofaf695.576

**Published:** 2026-01-11

**Authors:** Jesús Troya, Rafael Micán, María José Galindo, Otilia Bisbal, Lucía Romero, Miguel Torralba, Luis Buzón-Martín, Sara de la Fuente, Francisco Fanjul, Adrían Rodríguez, Alfonso Cabello, Isabel Sanjoaquin, Maria del carmen Navarro, María Aguilera, Carmen Hidalgo, Luis Enrique Morano, Clara Martínez, Inmaculada Poquet, Enrique Bernal, Ruth Caballero, Noemí Cabello, Juan Tiraboshi, María del Carmen Montero, María Jesús Vivancos, Francisco Tejerina, Guillermo Soria, Miguel Alberto de Zárraga, Mireia Cairó, Alberto Romero, Rebeca Cabo, Víctor Arenas, Maria Antonia Sepúlveda, Antonia Alcaraz, Cristina Escrich, Claudia González, Eva María Ferreira, Beatriz Valentín, Magdalena Muelas, Jara Llenas, Sara García, Juan Emilio Losa, Bárbara Alonso, José Sanz, Felix Gutiérrez, Nuria Vázquez, Juan José Corte, María Ángeles Garcinuño, Juan Carlos Gainzarain, Miriam Estébanez, Marisa Montes

**Affiliations:** Hospital Universitario Infanta Leonor, Madrid, Madrid, Spain; Hospital Universitario La Paz, Madrid, Madrid, Spain; Hospital Clínico de Valencia, Valencia, Comunidad Valenciana, Spain; Hospital Universitario 12 de Octubre, Madrid, Madrid, Spain; Hospital Universitario de Canarias, Santa Cruz de Tenerife, Canarias, Spain; Hospital Universitario de Guadalajara, Guadalajara, Madrid, Spain; Hospital Universitario de Burgos, burgos, Castilla y Leon, Spain; Hospital Universitario Puerta de Hierro, Madrid, Madrid, Spain; Hospital Universitario Son Espases, Palma de. Mallorca, Islas Baleares, Spain; Hospital Universitario Son Llatzer, Palma de mallorca, Islas Baleares, Spain; Hospital Universitario Fundación Jiménez Díaz, Madrid, Madrid, Spain; Hospital ClÃínico Universitario Lozano Blesa, Zaragoza, Castilla y Leon, Spain; Hospital Universitario Parc Taulí, Sabadell, Catalonia, Spain; Hospital Universitario de la Princesa, Madrid, Madrid, Spain; Hospital Universitario Virgen de las Nieves, Granada, Andalucia, Spain; Hospital Universitario Álvaro Cunqueiro, Vigo, Galicia, Spain; Hospital Clínico San Cecilio, Granada, Andalucia, Spain; Hospital de Denia Marina Salud, Alicante, Comunidad Valenciana, Spain; Reina Sofía General University Hospital, Murcia, Murcia, Spain; Hospital Universitario Miguel Servet, Zaragoza, Aragon, Spain; Hospital Clínico Universitario de Madrid, Madrid, Madrid, Spain; Hospital Universitario de Bellvitge, Bellvitge, Catalonia, Spain; Hospital Universitario de Torrejón, Torrejón, Madrid, Spain; Hospital Universitario Ramón y Cajal, Madrid, Madrid, Spain; Hospital General Universitario Gregorio Marañón, Madrid, Madrid, Spain; Hospital Universitario de Fuenlabrada, Fuenlabrada, Madrid, Spain; Hospital Universitario San Agustín, Gijón, Asturias, Spain; Hospital Universitario Mutua de Terrassa, Terrassa, Catalonia, Spain; Hospital Universitario de Puerto Real, Cádiz, Andalucia, Spain; Hospital Universitario Central de Asturias, Oviedo, Asturias, Spain; Hospital Universitario de Cabueñes, Cabueñes, Asturias, Spain; Hospital Universitario de Toledo, Toledo, Castilla-La Mancha, Spain; Hospital General Universitario Morales Meseguer, Murcia, Murcia, Spain; Hospital Verge de la Cinta de Tortosa, Tarragona, Catalonia, Spain; Hospital Universitario Marqués de Valdecilla, Santander, Cantabria, Spain; Complejo Asistencial de Segovia, Segovia, Castilla y Leon, Spain; Hospital Rí­o Hortega de Valladolid, Valladolid, Castilla y Leon, Spain; Hospital de Viladecans, Viladecans, Catalonia, Spain; Hospital de la Vega Baja, Alicante, Comunidad Valenciana, Spain; Hospital de Santa Caterina de Salt, Girona, Catalonia, Spain; Hospital Universitario de Alcorcón Madrid, Alcorcón, Madrid, Spain; Hospital Universitario Doctor José Molina Orosa, Las Palmas, Canarias, Spain; Hospital Universitario Prí­ncipe de Asturias, Alcalá de Henares, Madrid, Spain; Hospital General Universitario de Elche, Elche, Comunidad Valenciana, Spain; Complexo Hospitalario Universitario de Pontevedra, Pontevedra, Galicia, Spain; Hospital de Jove, Alicante, Comunidad Valenciana, Spain; Complejo Asistencial de Ávila, Ávila, Castilla y Leon, Spain; Hospital Universitario de Álava, Álava, Pais Vasco, Spain; Hospital Central de la Defensa Gómez Ulla, Madrid, Madrid, Spain; Hospital Universitario La Paz, Madrid, Madrid, Spain

## Abstract

**Background:**

Long-acting injectable cabotegravir and rilpivirine (LAI CAB+RPV) is a well-established regimen for people living with HIV (PLWH), offering high efficacy and tolerability. However, data are limited for individuals with a body mass index (BMI) > 30 kg/m², which may represent a potential risk factor for virological failure.

Survival curves for virological failure by baseline BMI category in patients on LAI CAB+RPV.

**Figure:**
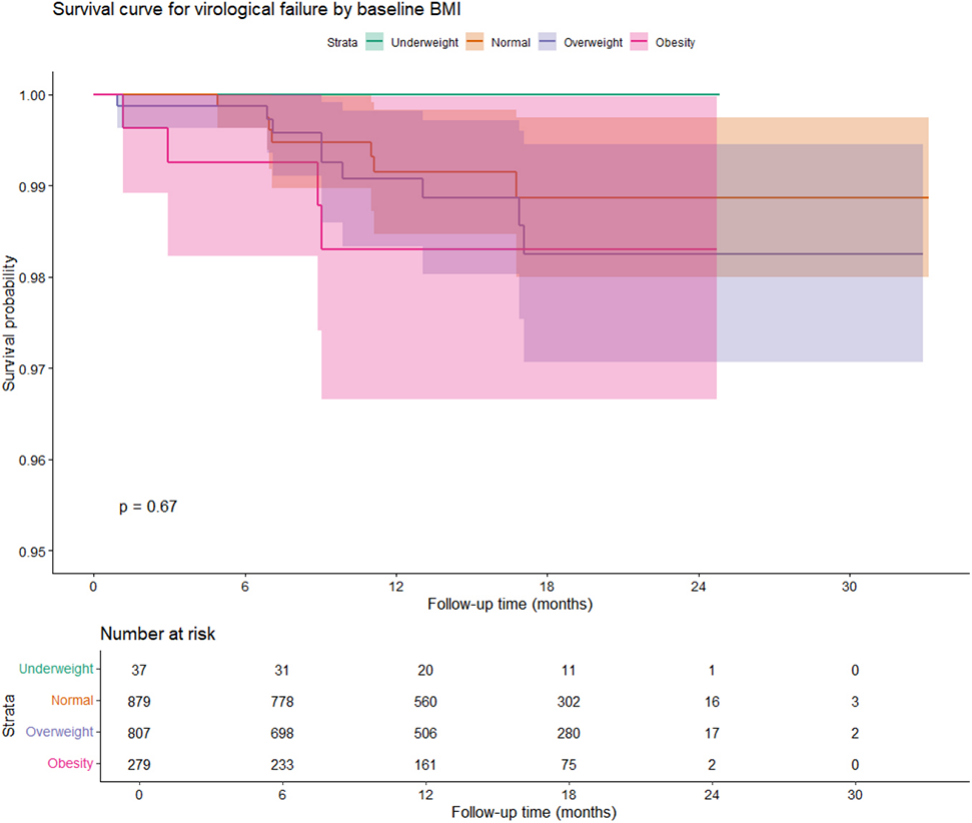

**Methods:**

We conducted a multicenter, retro-prospective study (RELATIVITY cohort in Spain) of virologically suppressed PLWH switching to LAI CAB+RPV, with a BMI > 30. We described this population and evaluated factors associated with virological outcomes using Kaplan–Meier analysis.

**Results:**

A total of 3,203 patients were included in the study: 37 (1.2%) were underweight, 879 (27.5%) had normal weight, 807 (25.2%) were overweight, and 279 (8.7%) were obese (BMI >30). The obese group was significantly older (47 vs. 44 years; p < 0.001) and had a higher proportion of women compared to the normal-weight group (22.3% vs. 14.5%; p < 0.001). Foreign nationality was also more frequent among obese individuals (33.6% vs. 27%; p = 0.028). Comorbidities increased significantly with higher BMI: hypertension (8.3% to 21.5%), diabetes (3.8% to 10.4%), dyslipidemia (17.6% to 36.2%), and non-alcoholic fatty liver disease (1.4% to 6.1%) (p < 0.001 for all comparisons).

The most frequent reasons for switching were treatment simplification (25.4%) and improvement in quality of life (55.6%). Standard needles were used in 68.7% of individuals with a BMI >30.

Kaplan–Meier analysis revealed no statistically significant differences in time to virological failure across BMI groups, although individuals with obesity showed a non-significant trend toward higher failure rates after 12 months. Cox regression analysis also confirmed the absence of an association. Four individuals (1.4%) in the obese group experienced virological failure, with emergent resistance mutations detected in only one case (L100I, K103N, L74M, T97A, G140S, Q148K, Q148R, E157Q).

Discontinuation rates were similar across groups, including those related to local (1.8%) and systemic (1.1%) adverse events.

**Conclusion:**

In real-world settings, LAI CAB+RPV appears to be a viable option for individuals with a BMI > 30 kg/m², demonstrating efficacy and safety rates comparable to those observed in other real-world cohorts.

**Disclosures:**

All Authors: No reported disclosures

